# Generalized and COVID related anxiety as risk factors for health outcomes among adolescents with HIV during COVID-19 in Tanzania

**DOI:** 10.21203/rs.3.rs-3921926/v1

**Published:** 2024-02-16

**Authors:** Hellen Siril, David Gitagno, Sylvia Kaaya, Matthew Caputo, Lisa Hirschhorn, Tumaini Nyamuhanga, Rachel Mtei, Charles Festo, Claudia Hawkins

**Affiliations:** Muhimbili University of Health and Allied Sciences; Muhimbili University of Health and Allied Sciences; Muhimbili University of Health and Allied Sciences; Northwestern University; Northwestern University; Muhimbili University of Health and Allied Sciences; Muhimbili University of Health and Allied Sciences; Management and Development for Health; Northwestern University

**Keywords:** Adolescents, HIV, COVID-19 pandemic, clinic visits, Viral suppression, Anxiety, Depression

## Abstract

The COVID-19 pandemic and associated prevention strategies caused widespread interruptions to care and treatment for people living with HIV. Adolescents living with HIV (AWHIV) were particularly vulnerable to poor mental and physical health during COVID-19. We assessed the burden of generalized and COVID-19-related anxiety and associations with adherence to HIV care and treatment and viral load suppression (VLS) among AWHIV during the peak of the COVID-19 pandemic in Tanzania.

**Methods:**

This cross-sectional study was conducted among AWHIV aged 15–19 years attending 10 clinics in Dar es Salaam from April 2022-February 2023. Study participants completed a self-administered questionnaire including Generalized Anxiety Disorder (GAD), COVID-19-related anxiety, and other psychosocial and physical health and support measures. HIV visit adherence, viral load and sociodemographic data were abstracted from patient health records.

**Analysis:**

Multivariable (MV) quasibinomial and logistic regression models examined associations of Generalized and COVID-19-related anxiety with visit adherence and HIV virologic suppression (HIV VL < 50 copies/mL). Data were analyzed using R software.

**Results:**

658 AWHIV (52% male) were included in this analysis. Most (86%) had been on antiretroviral treatment (ART) for at least four years, 55% attended at least 75% of their scheduled clinic visits, and 78% were HIV virologically suppressed. The median GAD and COVID-19-related anxiety scores were 2 (IQR: 0–5, and 26 (IQR: 13–43; respectively. Only 2% scored moderate-severe generalized anxiety (score 10–21). We found no significant associations between COVID-19-related anxiety or GAD and visit adherence. Higher GAD was inversely associated with VLS (adjusted odds ratio (AOR): 0.89 (95% CI 0.81, 0.98)). Female gender and higher quality of physical life were significantly associated with VLS.

**Conclusion.:**

Low levels of generalized and COVID-19 related anxiety were reported among Tanzanian AWHIV. Integrating screening and management of generalized anxiety screening into HIV care for AWHIV could improve VLS among this population.

## Background

Globally, the COVID-19 pandemic and responses to curb the spread of SARS-CoV-2 led to widespread interruptions to care and treatment for people living with HIV (PLHIV) [1, 2]. These disruptions had significant negative impacts on the wellbeing of adolescents living with HIV (AWHIV) who historically are more susceptible to anxiety and depression than older adults [3–5]. COVID-19 presented a number of additional environmental and social stressors such as isolation and abuse, worsening poverty and food insecurity, further exacerbating the risk of depression and anxiety and lowering physical well-being [8]. Disruptions to antiretroviral therapy (ART) access and support due to clinic shutdowns and staff shortage also exacerbated the risk of non-adherence to care and treatment among AWHIV during this time [9]. AWHIV in sub-Sahara African (SSA) and globally are known to have poor outcomes across the HIV care continuum, including low rates of viral suppression, adherence to treatment and retention in care [10–12].

In SSA, approximately six out of seven new infections occur among adolescents aged 15–19 and 100,000 in this age group are currently living with HIV in Tanzania [13, 14]. Between 10–27% of AWHIV in Tanzania have symptoms of depression and anxiety, rates that are significantly higher than in adolescents without HIV [15, 16]. AWHIV face a wide range of challenges that contribute to these conditions including high levels of stigma and discrimination and lack of social support, in addition to the challenges of normal adolescent development [6, 7]. Socio-cultural factors such as low socioeconomic status, childhood deprivation, and poor academic performance have been shown to contribute significantly to depression and anxiety among AWHIV in this setting [16]. In Tanzania, as in other neighboring East African countries, depression among AWHIV has been shown to have significant adverse effects on physical and psychosocial health and poor adherence to ART which in turn is associated with significant morbidity, mortality, lower quality of life and short life expectancy [17–21].

In Tanzania the government announced the first case of COVID-19 on March 16, 2020. Between March 2020 and April 29th 2022, over 33,904 people were confirmed with COVID-19 infection, and 803 deaths occurred from COVID-19-related complications [22]. Initial mitigation strategies introduced during COVID-19 included rapid implementation of non-biomedical prevention interventions including expanding of personal protection equipment (PPE), social distancing and a partial lockdown occurring between March to May 2020 [23]. Measures taken by the Tanzanian Government to prevent interruption to care among PLHIV included increasing the duration of prescriptions to reduce visit frequency and overcrowding in clinic waiting areas, and psychological support for adolescents on coping with COVID-19 and related fears. In July 2021, COVID-19 vaccines were introduced and by April 29th 2022, 3,915,900 of a population of 59 million were fully vaccinated [23].

Despite these interventions, it was widely reported that PLHIV in Tanzania had significant concerns and fear about their safety given their immunocompromised status, lack of clear information and feelings of being abandoned during the COVID-19 pandemic [24]. To date, the impact of the COVID-19 pandemic on AWHIV in Tanzania on generalized and COVID-19 related anxiety has not been assessed. It is also unknown whether these mental health conditions further affected AWHIV ability to adhere to ART, and routine care, independent of the pandemic itself. Exploring the potential impact of the COVID-19 pandemic on AWHIV, including their ability to remain in care and on treatment and effects on mental health, is important to understand so that culturally appropriate interventions at the individual and facility level to further support the care and mental health of AWHIV during future pandemics and other health system shocks.

## Methods

### Study aim

We assessed the burden of generalized and COVID-19–related anxiety among AWHIV during the peak of the COVID-19 pandemic in Tanzania and examined the association between these two anxiety measures and adherence to HIV clinic visits and viral suppression (VLS). We hypothesized that higher levels of self-reported generalized and COVID-19 related anxiety were associated with higher rates of nonadherence to care and lower rates of VLS.

### Design

This cross-sectional study was conducted between April 2022 and January 2023.

### Study Setting

The study was conducted in 10 HIV Care and Treatment Centers (CTCs) in Dar es Salaam city, Tanzania. The CTCs were high volume facilities with at least 5000 people PLHIV enrolled in ART services.

### Study population

Participants were AWHIV ages 15–19 that were active in care as of September 2019. Active in care was defined as attending all scheduled visits (+/−three days) for 12 months prior to September 2019.

#### CTC care for AWHIV

AWHIV attend CTCs every one, three, or six months depending on how clinically stable they are per the 2018 Tanzanian HIV care and treatment national guidelines [25]. Visits are scheduled on Saturdays to allow AWHIV at school or work to attend on non-weekdays. During CTC visits, AWHIV receive comprehensive HIV services including health education on HIV prevention, sexual and reproductive health services, ART initiation and counseling, peer-to-peer adherence support, and linkages to other health services when necessary. To support wellness and adherence, AWHIV can attend adolescents’ camping quarterly which include 3–5 days of sports, testimonies and discussions of some of the challenges that AWHIV face adhering to ART, coping with school, society, puberty, stigma, and HIV status disclosure issues. AWHIV with high HIV viral loads (VL > 1000 copies/ml) receive additional interventions including Enhanced Adherence Counselling sessions.

## Study procedures and data collection

### Sample size calculation

We based our power calculations on known population means of the Swahili-translated version of the GAD-7 in a sample of Kenyan adults living with HIV [26]. With an alpha level of 5% (0.05) and a beta level of 20% (0.20), we needed to enroll between 670–680 individuals to achieve 80% power to detect a small effect size between viral load (VL) suppression and GAD scores.

### Selection of study participants

Participants were randomly selected from an electronic CTC2 clinical database available at each of the participating sites. We sampled 676 AWHIV and surveyed 658 who were able to be reached by phone and provide consent. 18 AWHIV were removed from the study because they couldn’t be reached by phone or did not consent to being in the study.

### Consenting procedures

All participants provided a written informed consent, which was obtained from literate participants ≥ 18 and legal guardian(s) for participants < 18 yrs. Participants ≥ 18 and those 15–17 years who came alone to the clinic, were married, and classified by the clinic as emancipated minors provided informed consent on their own. Participants aged 15–17 years, who came with parents/guardians to the clinic, provided assent to participate after their parents /guardians provided signed informed consent.

#### Data collection

After providing consent, eligible participants completed self-administered questionnaires in Swahili including: Generalized and COVID-19-related anxiety, depression, social support, stigma, self-management, and physical health (Appendix 1–2). Additional data were collected in the survey on sociodemographic characteristics (age, sex, marital status, education level, residential district, occupation, employment or school status). HIV-related data was abstracted from participant records and included date of HIV diagnosis, duration on ART, and most recent HIV viral load results, and visit dates. Study data were collected and managed using Research Electronic Data Capture (REDCap) at Muhimbili University of Health and Allied Sciences.

## Survey measures

**Generalized anxiety disorder (GAD)** symptoms were assessed using the GAD-7 item self-report screening scale [27] which has been validated previously in South Africa and Kenya [26, 28]. GAD-7 assesses past two-week symptoms of Generalized Anxiety Disorder based on the Diagnostic and Statistical Manual version 4. A four-point Likert scale provides scores of 0–3 for responses to each item as “not at all”, “on several days”, “on more than half the days”, and “nearly every day”, with a summed scores range of 0–21. Summed scores were categorized as minimal symptoms (0–4), mild symptoms (5–9), moderate symptoms (10–14) and severe symptoms (15–21).

**COVID-19 related anxiety** was assessed using a structured questionnaire from Turkey [29]. Questions included COVID-19 knowledge, precautions, and potential changes of behavior that could negatively impact ART adherence during COVID-19 pandemic. Responses were recorded on a Likert scale from 1 (minimum) to 10 (maximum) for each question with a summed score range of 6–60.

**Self-management (SM)** was assessed using the Adolescents HIV Self-Management (AdHIVSM) tool, developed in South Africa [30]. The AdHIVSM tool has 35-item measure of five components of AdHIVSM including believing and knowing, goals and facilitation, participation, HIV biomedical management, and coping and self-regulation with good validity and reliability for this population [30]. Items are scored using a four-point Likert scale with item scores ranging from strongly agree to strongly disagree and a summed score range of 21–105.

**Perceived social support** was measured using the 12-item Multidimensional Scale of Perceived Social Support (MSPSS) [31], a 12-item scale developed for use in older adolescents and emerging adults, with three dimensions: significant Other, Family, Friends each comprising four item subscales of the instrument. Response options range from 1–7 (from very strongly disagree to very strongly agree) preventing against ceiling effects with a summed possible scores range of 32–84. The MSPSS has been validated for use in the East and Southern African context [32, 33].

**HIV Stigma** was assessed using the 12-item version of the Berger HIV-related stigma scale with four dimensions: (1) personalized stigma; (2) disclosure concerns; (3) negative self-image and (4) concerns with public attitudes, each comprising a subscale of the instrument with a summed possible score range of 12–48. This tool has been validated in Swahili in Tanzania [34].

### Depression.

Depression was measured using the Patient Health Questionnaire version 9 (PHQ-9), which has been validated in Tanzania [35, 36]. The PHQ-9 is a nine-item scale rated on a Likert-type scale ranging from 0 ‘not at all’ to 3 ‘nearly every day.’ Item scores are summated to derive a total score ranging from 0 to 27.

### Quality of physical health.

This was measured by the World Health Organization’s quality of life measure for HIV (WHOQoL-BREF-HIV) [37], using the physical functioning quality of life domain. It rated on a Likert-type scale ranging from1 not at all to 5 an extreme amount, with a summed scores range of 4–25.

## Survey questionnaire translation and cultural adaptation

All survey measures were translated and culturally adapted using an established process to ensure clarity of the questions among Tanzanian AWHIV [35]. Two native speakers of Tanzanian Kiswahili who were conversant with the project independently translated the questions from English to Kiswahili (Kiswahili versions 1). An independent team of two native speakers of Tanzanian Kiswahili, not conversant with the study performed a back translation of the two Kiswahili versions to English. This was followed by a review by a panel of experts including a Kiswahili linguist, four translators, a social worker, and the study team, of all the discrepancies between items in the original English and the two English back-translations from Kiswahili. This step was to ensure items and their related concepts have Kiswahili conceptual equivalence. This review exercise resulted in a Kiswahili Version 2 of the measures of interest.

A panel of 12 adolescents was interviewed using the questions in the Kiswahili version 2 tools (6 Males and 6 Females of age range 15–19) and cognitive interviews were conducted immediately after the survey to explore the AWHIV thinking process when responding to each item. Each item was read aloud to participants, and probes explored how participants had arrived at this response asking them to try to recall what they had been thinking prior to responding, how relevant the item was to their reality and lives, and how they think other people aged 15–19 years living with HIV would have understood the item. To capture the perspectives of adolescents verbatim, cognitive interviews were audio-recorded and transcribed. The feedback from the cognitive interviews was used to finalize a Kiswahili version 3 which was piloted before initiating data collection for this study.

## Data analyses

### Primary Outcome definitions

We calculated HIV VLS as a having viral load of less than 50 copies per ml at the last visit before the study visit. Visit adherence was defined as the proportion of scheduled clinic visits attended in the past 24 months prior to 30th December 2021. Visit adherence was treated as both a binomial variable and as proportions categorized as < 25%, > 25- ≤50%, > 50-≤75% and > 75%.

### Statistical analysis

Descriptive statistics (counts and proportions for categorical or median and interquartile range (IQR) for continuous data) were calculated for all demographic, clinical and outcome variables. Multivariable quasibinomial and logistic regression models were generated for the visit adherence and virologic suppression outcomes, respectively. Demographics of interest (sex, age, education, and residing with parents), time on ART, and psychological outcomes (perceived support, stigma, self-management, depression, COVID-related anxiety, generalized anxiety, and quality of physical life) were considered for covariates in both models. These variables were selected under the belief that they were important to adjust for or could be associated with our outcomes based on previous literature. The number of covariates was limited so that there would be at least 10 events per variable [38]. A quasibinomial model for adherence was determined necessary to account for overdispersion, which was confirmed by the dispersion parameter in the final model. All patients were assigned equal weight in the adherence model, regardless of their number of scheduled visits. Patients who reported being on ART for less than one year were excluded from the logistic regression model for virologic suppression. Linearity between continuous covariates and the logits of the outcomes was assessed graphically. Multicollinearity was assessed with generalized variance inflation factors (GVIFs), with any (GVIF^1/(2DF)^)^2^ > 3 raising consideration for removal. Observations were excluded from each respective model if data were missing for any of the included covariates or outcomes. Data analysis was performed in Stata version [39] and R 4.2.3 [40].

## Results

### Baseline characteristics of study population

There were 658 AWHIV (52% male) included in this analysis. The majority (84%) were from larger health centers in Ilala (31.5%), Temeke (27.5%), and Kinondoni (25%), and 77% had attained an ordinary level or advanced secondary school education (Table 1). Most (88%) participated in a youth peer pairing intervention routinely delivered in HIV CTCs to help adolescents living with HIV stay on their medication and in care; and 86% had been on ART for at least four years. Just over half (55%) attended at least 75% of their scheduled clinic visits with a median (IQR) adherence of 80% (64%-92%), and 77% were HIV virologically suppressed (HIV VL < 50 copies/mL).

**Figure 1:**
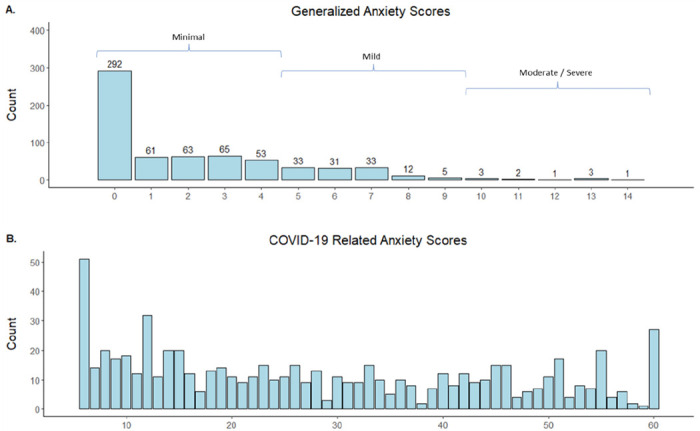
Distribution of COVID-related (A) and Generalized anxiety (B) scores among participants A. Distribution of summed scores from GAD-7 instrument, with summed score values on the horizontal axis and frequencies on the vertical axis. Summed scores were calculated as the sum of 7 Likert scale items scored from 0-3, resulting in a summed score range of 0-21. Clinical categorizations of anxiety levels based on scores are also indicated. N = 658. B. Distribution of summed scores from COVID-19 related anxiety questionnaire, with summed score values on the horizontal axis and frequencies on the vertical axis. Summed scores were calculated as the sum of 10 Likert scale items scored from 1-10, resulting in a summed score range of 6-60. N = 658

Participants scored a median GAD score of 2 (IQR: 0–5), the scores distribution had one peak at the minimum possible scores (Fig. 1A) with 81% of participants scoring at minimal while 2% scored at moderate-to-severe generalized anxiety (scores of 10–21) (Table 1). The median COVID-19-related anxiety score was 26 (IQR: 13–43), with scores following a bimodal distribution with peaks at the minimum and maximum possible scores (Fig. 1B). The highest scoring COVID-19-related anxiety subdomain, with a median score 5 (IQR: 2–10) was ‘The *likelihood of a country running short of ARVs due to disruption of production’* (Table 2). Participants reported a median PHQ-9 score of 2 (IQR: 0–5) with 25% of scores reflecting mild and 5.4% reflecting moderate-severe symptoms of depression. The median score for perceived social support was 68 (IQR: 60–76); and the highest median subscale score was 26 (IQR: 24–28) for family support. The median stigma score was 26 (IQR: 21–30), and the highest median subscale score was for disclosure concerns with a median score of 9 (IQR: 9–11). The median scores for self-management and quality of life were 86 (IQR: 77–95) and 15 (IQR: 13–16), respectively (Table 2).

#### Factors associated with visit adherence and HIV VLS.

##### Regression variable selection and model diagnostics

Of the 658 participants, 619 (94%) were included in the multivariable quasibinomial regression model for visit adherence, with 39 removed due to missing values. A total of 515 (78%) participants were included in the multivariable logistic regression model for virologic suppression, with 140 removed due to missing values for viral load. The stigma variable violated the assumption of linearity with log odds for both models and was removed. All other continuous covariates satisfied the assumption of linearity with log odds and no substantial multicollinearity was found in either model.

##### Visit adherence

After adjusting for sociodemographic, clinical, and HIV treatment-related factors, there was no association between COVID-19-related anxiety (AOR = 1.00 (95% CI 1.00, 1.01); p = .862) or GAD (AOR = 1.01 (0.98, 1.05); p = .468) and visit adherence. Females were marginally more likely to adhere to visits than males (AOR =1.18 (1.00, 1.40); p = .055), though this result was not statistically significant. All other factors showed no significant associations with visit adherence (Table 3).

##### HIV *VLS*

After controlling for sociodemographic, clinical, and HIV treatment-related factors, no associations were observed between COVID-19-related anxiety (AOR = 1.01 (1.00, 1.03); p = .114) or depression (AOR = 1.01 (0.93, 1.09); p = .869) and VLS. Higher GAD however was associated with lower odds of VLS (AOR = 0.90 (0.82, 0.98); p = .021). Higher quality of physical life (AOR = 1.16 (1.04, 1.30); p = .011) and female sex (AOR = 1.56 (1.00, 2.43); p = .049) were associated with higher odds of VLS. A association was found between VLS and living with one’s parents although this was not significant (AOR = 1.56 (.99, 2.44); p = .055). There were no significant associations between VLS and perceived support, self-management, or any other covariate (Table 4).

## Discussion

In this Tanzanian study exploring COVID-related and generalized anxiety and its association with health outcomes among ALWH in care, we observed overall low proportions of moderate-severe generalized and COVID-19 specific anxiety. Importantly, there was no association between COVID-19 anxiety and either visit adherence or VLS. However, higher GAD was associated with lower odds of VLS, after adjusting for multiple factors. Maintaining VLS is critical to reducing transmission and HIV-related morbidity and mortality in this vulnerable population.

The low rates of both COVID- related and generalized anxiety among ALWH during COVID-19 in this study were surprising given the multiple challenges and widespread disruptions caused by the pandemic globally [41]. Several countries in SSA as well as globally have reported adverse effects of the COVID-19 pandemic on health care services, the environment and social support networks in adolescents with and without HIV [42, 43]. In a study conducted by the Adolescent HIV Prevention and Treatment Implementation Science Alliance (AHISA) in 2021, teams from multiple countries in SSA reported interruptions to prevention programs, diagnostic testing, and access to ART during COVID-19. Individual-level impacts included feelings of social isolation, loneliness, loss to follow-up, food insecurity, poverty, and increases in adolescent pregnancies and sexually transmitted infections [10]. Many studies have also reported an increased risk of anxiety, depression, feeling lonely, and a reduction in the quality of life among adolescents during the COVID-19 lockdown because of these stressors [44–46].

There are several possible explanations of the lower rates of COVID-19 related anxiety in our study including low perceived risk of COVID-19, high levels of social support and short periods of lockdown in Tanzania which could have prevented isolation and anxiety as a result. It is notable that the highest COVID-19 anxiety score in our study was in the individual domain *‘The likelihood of a country running short of ARVs due disruption of production’* suggesting adolescents are more concerned about care interruptions than the risk of COVID-19 to themselves. In contrast, the ratings for self-perceived risk from COVID-19 infection were low. This concern has been reflected in other studies from Tanzania and other settings and is being explored in ongoing qualitative work by our team. In a survey of COVID knowledge and risk perception conducted among Tanzanians > 18 during the COVID pandemic, the proportion of persons who perceived themselves as low risk for COVID-19 was significantly higher than those who perceived themselves as high risk. Also, 44% believed the hot climate prevented COVID-19 spread [47]. In a study in Lebanon during COVID-19, among the 18% of adolescents who were found to have severe social anxiety, no correlation was found between having anxiety and acknowledging or fearing COVID-19 morbidity [48].

The high rates of perceived social support, parental support and other socio-environmental factors reported among study participants, all of which are strongly associated with positive mental health in AWHIV, could have also contributed to low rates of both COVID-19 related anxiety and GAD [49, 50]. In a recent systematic review of mental health conditions among AWHIV by Too et al., higher social support, family cohesion and positive parenting were all associated with lower rates of anxiety and depression [49]. This high level of social support could have also contributed to the low rates of depression observed in our cohort, a mental health condition highly correlated with anxiety in this population [51, 52].

Lockdowns introduced by COVID-19 severely restricted social interactions and several studies have reported the negative impacts of lockdown on adolescent mental health including feelings of isolation and separation from family and friends [53, 54]. In Tanzania, stringent nationwide lock downs introduced during COVID-19 were significantly shorter than in other countries (2–3 months) [22, 55 56]. Disruptions to social support systems and levels of anxiety among AWHIV as a result, could have been minimized and support from the family or other non-healthcare related support may have also remained constant or even increased. This high level of support and continuity of care could have also contributed to the low rates of HIV stigma observed in this study population.

Although we did not observe any association between COVID-19 related anxiety or GAD and visit adherence in this study, it is notable that visit adherence overall was < 75% in almost half of the study population. This is likely due to the impact of the COVID-19 pandemic on routine HIV services which even in the absence of long lockdowns were severely disrupted [57].

Despite suboptimal visit adherence, over 80% of participants were virologically suppressed. Both low GAD and high physical health scores were associated with VLS in this study. In Tanzania, during COVID-19, the government allowed the dispensing of extra ART to minimize physical contact which likely protected against significant interruptions to treatment and declining rates virologic suppression as a result. Our findings are similar to those from the Congo and other SSA countries which also showed stable or improved VLS during COVID-19 for many of the same reasons [58–60]. High ratings in social support, and self-management also likely contributed to the high proportion with VLS, although associations between these factors and VLS were not significant after adjusting for other potential confounders. High levels of social support and high self-management among AWHIV have also been shown to be associated with improved outcomes in HIV in several other studies [61, 62].

Finally, female gender was also marginally associated with improved visit adherence (p = .055) and associated with VLS (p = .049), which has also been reported before the COVID-19 pandemic [60]. It is an important finding to note considering the disproportionate effect COVID-19 had on adolescent girls mental health and well-being generally, and increased exposure to gender-based violence, school exclusion and economic hardships [63, 64].

To our knowledge this is one of the largest studies examining COVID-19 related anxiety and its association with health outcomes in AWHIV in Tanzania. However, our study had some limitations. The COVID related anxiety questionnaire adopted was one of the few published scales at the time of the study, and had not been validated in Tanzania or other LMIC settings during the early phases of the pandemic. Questions mostly focused on perceived risk of COVID-19 and concerns about COVID-19 transmission. The survey did not include questions on impacts on health and memory; financial wellbeing and lifestyle; social support; general health; coping strategies; and self-care. However, many of these areas were assessed in other validated questionnaires that were administered as part of this study which provided important insight into these areas in times of stress even if the questions were not COVID-19 specific. This study was also performed after COVID-19 surveillance and mitigation measures in Tanzania were halted in June 2020, only 3 months after the onset of the pandemic. Thus, these findings may not necessarily be generalizable to other settings, where mitigation measures were more stringent and general awareness of COVID-19 perhaps higher. Given the dynamic situation of COVID-19, prompting a range of different responses from individual to policy levels, the results may not be reflective of other times during the pandemic.

## Conclusion

Our study found low levels of COVID- 19 related anxiety among Tanzanian AWHIV, which did not adversely impact treatment outcomes during the pandemic. The inverse association observed between GAD and VLS suggests a need for targeted anxiety screening and interventions to improve ART adherence in adolescent clinics and continuity of these services especially in times of stress. Interventions focused on improving physical health and supporting family and social support networks can also help optimize HIV health and outcomes in this vulnerable population.

## Figures and Tables

**Table 1:** Baseline characteristics of 658 Adolescents living with HIV in Tanzania enrolled in the study

Variable	n (%) or median (IQR) N = 658
Gender	
Male	340 (51.70)
Female	318 (48.30)
Age group (Years)	
> 15-≤18	415 (63.10)
> 18-≤19	243 (36.90)
Persons in household	
Parents	430 (65.40)
Grandma/pa	162 (24.60)
Other relatives	50 (7.60)
Orphanage Centre	12 (1.80)
Live alone	4 (0.60)
Education	
No school or primary school only	152 (23.10)
Ordinary or advanced secondary school	506 (76.90)
Occupation	
Student	421 (64.00)
Self-employed/Employed	185 (28.10)
Unemployed	52(7.90)
Attendance in:	
Youth Camp	4 (0.60)
Saturday Youth Clinic	16 (2.40)
Multidisciplinary (MDT) Professional Clinic	58 (8.80)
Youth peer pairing	580 (88.20)
District	
Ilala	207 (31.50)
Kinondoni	165 (25.10)
Temeke	181 (27.50)
Ubungo	72 (11.00)
Kigamboni	33 (5.00)
Scheduled Visits	11 (7, 15)
Adhered Visits	8 (5, 12)
Visit adherence	0.80 (0.64, 0.92)
< 25%	12 (1.90)
>25-≤50%	68 (11.00)
>50-≤75%	199 (32.10)
> 75%	340 (54.90)
Missing	39
Duration on ART (months)	
<=1 year	10 (1.60)
1–2 years	45 (7.20)
3–4 years	33 (5.20)
4 + years	541 (86.00)
Missing	29
VL suppression(copies/ml)	
<1000	454 (87.60)
>=1000	64 (12.40)
Missing	140
VL suppression(copies/ml)	
<50	403 (77.80)
>=50	115 (22.20)
Missing	140

IQR: Interquartile range, ART; antiretroviral therapy, VL: HIV viral load

**Table 2:** Psychosocial outcomes of 658 ALWH

Outcome (score range)	n (%) or median (IQR) N = 658
Generalized anxiety^6^	
Total score (0, 21)	1 (0, 4)
Minimal (0–4)	534 (81.2)
Mild (5–9)	114 (17.3)
Moderate/Severe (10–21)	10 (1.5)
COVID-19 anxiety	
Total score (6–60)	26 (13, 43)
Spread of COVID-19 in Tanzania (1–10)	3 (2, 8)
The risk of acquiring covid-19 (1–10)	4 (2, 9)
The risk of acquiring covid-19 and becoming seriously sick (1–10)	4 (1, 9)
The likelihood of a country running short of ARVs due disruption of production (1–10)	5 (2, 10)
The risk of poor adherence to ARVs in the era of COVID-19 (1–10)	3 (1, 7)
The risk of transmitting COVID-19 virus to another person (1–10)	3 (1, 8)
Depression^1^	
Total score (0–21)	2 (0, 5)
None (0–4)	458 (69.6)
Mild (5–9)	165 (25.0)
Moderate (10–14)	30 (4.6)
Severe (15–21)	5 (0.8)
Perceived social support^2^	
Overall (32–84)	68 (60, 76)
Significant others (4–28)	24 (22, 28)
Family (4–28)	26 (24, 28)
Friends (4–28)	19 (13, 24)
Self- management^3^	
Overall (21–105)	86 (77, 95)
Believing and knowing (7–24)	23 (21, 24)
Goals and facilitation (6–24)	23 (20, 24)
Participation (5–27)	23 (20, 27)
Biomedical management (0–15)	7 (5, 11)
Copying and Self-regulation (0–15)	12 (9, 15)
Stigma^4^	
Overall (12–48)	26 (21, 30)
Personalized stigma (3–12)	4 (3, 6)
Disclosure concerns (3–12)	9 (9, 11)
Concerns about public attitudes (3–12)	6 (3, 7)
Negative self-image (3–12)	5 (3, 7)
WHOQOL Physical Health^5^ Domain	
Overall (7–16)	15 (13, 16)

WHOQOL; World Health Organization Quality of Life

**Table 3:** Factors associated with adherence to visits among study participants

	Visit Adherence			
	Crude OR	Crude p	Adjusted OR	Adjusted p
Gender: Male	Ref	Ref	Ref	Ref
Gender: Female	1.18 (1.00, 1.38)	.049	1.18 (1.00, 1.40)	.055
Age: 15–17	Ref	Ref	Ref	Ref
Age: 18–19	1.00 (0.85, 1.19)	.984	1.01 (0.85, 1.20)	.903
Ordinary/Secondary School	Ref	Ref	Ref	Ref
No School	1.07 (0.88, 1.30)	.487	1.14 (0.93, 1.40)	.217
Live without parents	Ref	Ref	Ref	Ref
Live with parents	1.06 (0.89, 1.25)	.522	1.02 (0.86, 1.21)	.832
Time on ART	1.01 (0.99, 1.03)	.180	1.01 (1.00, 1.03)	.144
Perceived Support	1.01 (1.00, 1.01)	.166	1.00 (0.99, 1.01)	.631
Self-Management	1.00 (1.00, 1.01)	.510	1.00 (0.99, 1.01)	.962
Depression	0.98 (0.96, 1.00	.073	0.98 (0.95, 1.01)	.247
Covid Anxiety	1.00 (0.99, 1.00)	.827	1.00 (1.00, 1.01)	.816
Generalized Anxiety	1.00 (0.97, 1.00)	.758	1.01 (0.98, 1.05)	.470
Quality of Physical Life	1.03 (0.99, 1.07)	.127	1.03 (0.99, 1.08)	.152

OR: Odd ratio, ART: antiretroviral therapy

**Table 4:** Factors associated with Viral Load suppression among study participants

	VL Suppression Logistic Regression Model			
	Crude OR	Crude p	Adjusted OR	Adjusted p
Gender: Male	Ref	Ref	Ref	Ref
Gender: Female	1.31 (0.86, 1.99)	.207	1.56 (1.00, 2.43)	.049
Age: 15–17	Ref	Ref	Ref	Ref
Age: 18–19	1.21 (0.78,1.88)	.389	1.15 (0.73, 1.81)	.548
Ordinary/Secondary School	Ref	Ref	Ref	Ref
No School	0.79 (0.49,1.28)	.336	0.93 (0.55, 1.58)	.795
Live without parents	Ref	Ref	Ref	Ref
Live with parents	1.49 (0.97,2.29)	.066	1.56 (0.99, 2.44)	.055
Time on ART	1.01 (0.96,1.07)	.598	1.01 (0.96, 1.07)	.615
Perceived Support	1.02 (1.00,1.04)	.105	1.02 (0.99, 1.04)	.179
Self-Management	1.00 (0.99,1.02)	.804	0.98 (0.96, 1.01)	.142
Depression	0.94 (0.89, 1.00)	.061	1.01 (0.93, 1.09)	.869
Covid Anxiety	1.00 (0.99,1.02)	.633	1.01 (1.00 1.03)	.114
Generalized Anxiety	0.91 (0.85,0.98)	.012	0.90 (0.82, 0.98)	.021
Quality of Physical Life	1.16 (1.05,1.28)	.003	1.16 (1.04, 1.30)	.011

VL: viral load, OR: Odd ratio, ART: antiretroviral therapy

## Data Availability

**D**ata supporting the results reported in this study can be available in the study database stored at MUHAS REDCap account by requesting from the principal investigator.

## Availability of Data and Materials:

The data that support the findings of this study are available from the authors but restrictions apply to the availability of these data, which were stored in REDCap database saver for under the license of Muhimbili University of Health and Allied Sciences(MUHAS) and therefore are only available on request (neemasiril@gmail.com).

## Abbreviations

If abbreviations are used in the text they should be defined in the text at first use, and a list of abbreviations should be provided.

AdHIVSM: Adolescents HIV Self-Management tool
AHISA: Adolescent HIV Prevention and Treatment Implementation Science Alliance
AOR: Adjusted Odds ratio
ART: Antiretroviral Therapy
AWHIV: Adolescent living with HIV
COVID: Corona virus Disease of 2019
CTC: Care and Treatment Clinic
GAD: Generalized Anxiety Disorder
GVIFs: Generalized variance inflation factors
HIV: Human Immunodeficient Virus
IQR: Interquartile range
LMIC: Low- and Middle-Income countries
MSPSS: Multidimensional Scale of Perceived Social Support
PHQ: Patient Health Questionnaire
PLHIV: People living with HIV
SSA: Sub Saharan Africa
VL: Viral Load
VLS: Viral Load suppression
WHOQoL-BREF-HIV: World Health Organization Quality of Life brief questionnaire for persons living with HIV
